# Selections and Abstracts

**Published:** 1916-02

**Authors:** 


### SELECTIONS AND ABSTRACTS

### HERMAPHRODITISM.

(The Medical Times.)

Blair Bell of London, recently presented before the Obstetrical and Gynecological section of the Royal Society of Medicine a case of the so-called "true hermaphroditism." This is so rare a condition that great interest attaches to the paper, which is published in full, with many illustrations, in the Proceedings: True hermaphroditism in man is included in the variety known as "glandular partial hermaphroditism." All the recorded cases except one of glandular partial hermaphroditism which may be accepted as authentic—and there are only four other possible cases in addition to Bell's—have been found to possess mixed gonads, so-called ovo-toestes, with or without irregularities in the sex characterization of the genital ducts, external genitalia and secondary characteristics.

In 1873 Klebs, in his classical paper on the subject, claimed that "true hermaphroditism," as he called it, might occur in the following varieties in man:

(1) Bilateral hermaphroditism, in which an ovary and testis are present on both sides.

(2) Unilateral hermaphroditism, in which there is an ovary or testis on one side, and an ovary and testis on the other.

(3) Lateral hermaphroditism, in which an ovary is present on one side and a testis on the other.

This classification has been adopted by many subsequent writers and it is supported by Blacker and Lawrence who in 1896 reported the first case of ovo-testis, or combined ovary and testis. They investigated the literature up to that date, and came to the conclusion that of the enormous number of supposed cases of what was called "true hermaphroditism" there was only one—their own—recorded of the unilateral variety, after the classification of Klebs; and that there was possibly one—Heppner's, recorded in 1870—of the bilateral variety, and two—recorded by Schmorl and Obolonsky in 1888—of the lateral. The evidence in regard to these, except possibly to Blacker's and Lawrence's cannot, however, be considered satisfactory, and it has been demolished by Meixner and others. Tuffier and Lapointe, also, in their paper published in 1811, apparently accept only the case of Blacker and Lawrence of those published prior to 1897; but they add to this case those of von Salen, Garre, Landau and Pick, and Schickele, recorded subsequently to Blacker's and Lawrence's paper.

Pick, in an important paper published in 1914, discusses exhaustively the question of this so-called "true hermaphroditism," and comes to the same general conclusions of this variety reported and accepted should be called "glandular partial hermaphrodites." This author records five cases of ovo-testis occurring in pigs, all of which he had himself examined. Pick also states that only four genuine cases had been recorded in man up to the date of his paper. Of those already mentioned apparently he admits only the case of von Salen, but he adds those of Simon, Uffreduzzi, and Gudernatsch. Foster has recently recorded a similar case, which, however, cannot be accepted as no histological details are given.

Bell's case S. P., aged 17, was first seen by him November 8, 1912.

Past history: Menstruation had commenced during her fourteenth year. There was no menstrual pain. The patient was said to have suffered from inflammation of the bowels when she was 7.

Present history: She has had amenorrhoea for eighteen months, and there have been no menstrual molimina. The general health is good. There has been no trouble with the bowels or bladder. The voice has been getting deeper.

On examination nothing abnormal was felt in the abdomen or per rectum. The thyroid was found to be slightly enlarged. A diagnosis of suprarenal hyperplasia was made, and the patient was treated with ovarian and thyroid extracts for some time.

On August 25, 1914, the patient, who had not been seen for eight months, again presented herself. It was then at once noticed that she had become more masculine in appearance. She had a slight mustache and a masculine distribution of the hair on the body. There was still complete amenorrhoea.

The patient was examined under an anesthetic a few days later. The clitoris was found to be much enlarged, measuring 2 inches in length, and there was a well-marked prepuce. Per vaginam, the left genital gland could be felt somewhat enlarged. No tumor was discovered in the suprarenal region. Subsequently, the patient was admitted to the hospital. She was then 19, and the amenorrhoea had lasted for over three years.

On September 3, 1914, the abdomen was opened in the middle subumbilical line. The left genital gland was found to be the size of a plum. It had a very smooth surface and resembled a testis; the superficial blood-vessels were injected, especially in the neighborhood of the hilum. No adhesions were present. A wedge-shaped piece was removed lengthwise from the convexity of the organ for histological examination. When cut into for the removal of this piece of tissue the organ presented a yellow, fatty appearance. The raw surfaces were brought together with a cutgut suture. The genital gland on the right side appeared to be a normal, somewhat small ovary. A piece was removed for section in a similar manner to that adopted in the case of the left genital gland. Finally, a small graft from an ovary removed from a patient operated upon a few minutes before was implanted in the uterus. The suprarenal regions were palpated with the hand in the peritoneal cavity, but beyond a slight rough feeling in the neighborhood of the left, which might have been due to the head of the pancreas, nothing abnormal was discovered.

The report from the Pathological Laboratory on the pieces excised was as follows: "Right ovary: Section shows an ovary, the stroma of which consists of very dense connective tissue, but ovulation has taken place, there being present a large corpus luteum and the scars of the corpora lutea, and also an almost mature Graafian follicle. Left ovary: Section shows what is undoubtedly a columnar-celled carcinoma with well-marked acini."

Placing an unwise faith in this report, I reopened the abdomen on September 22, 1914, and removed both ovaries, the tubes, and the fundus of the uterus. The patient made a good recovery, but subsequently suffered from slight menopausal symptoms.

When I came to examine the sections myself I came to the conclusion that the left genital gland was, in my opinion an ovo-testis, and not the seat of malignant growth as reported. This opinion was afterwards confirmed by the report of the Pathological Reference Committee of the Liverpool Medical Institution.

The specimen removed consists of the fundus uteri, the Fallopian tubes and the genital glands, each in the position normal to the female and attached to the uterus by an "ovarian" ligament.

A laryngeal examination was as follows: "The larynx appears to present definitely male characteristics; the cavity is very roomy and the vocal cords are both broader and larger than those of the ordinary female larynx. Using a graduated laryngeal mirror, I attempted to measure their length and, although it is difficult to do this with much accuracy owing to the distance between the mirror and the objects reflected in it, I was able to satisfy myself that they measured not less than 24 mm., which is scarcely below the average in the male. The breadth appeared to be nearly one and a half times what is usual in the female. The epiglottis and arytenoids are large for a female, but I think less strikingly so than the true cords and the laryngeal cavity."

The histological findings in this case are probably typical of glandular hermaphroditism, in which there is an ovo-testis on one side only.

The right genital gland is a normal ovary, although small and not in a particular active state. There are to be seen in section a mature Graafian follicle and small hyaline bands in the ovarian stroma, the remains of corpora albicantia.

The histological appearance of the left organ present quite a different picture. A low power view shows that the central portion which forms nearly the whole of the organ, is made up of what, in the first instance, was supposed by the pathologists to be a malignant neoplasm. A thin capsule of normal ovarian tissue—Graafian follicles, primordial ova and stroma covered in places by capsular epithelium—surrounds this central portion.

The nature of the central part of the organ is, of course, the interesting feature of the case. It consists of tubules lined for the most part with several layers of columnar cells, although in places the tubules ar eseen to be lined with one layer of epithelium, and sometimes to be widely distended with secretion of irregular masses of similar cells and, most important of all, of a large number of interstitial cells which are eosinophile, and resemble exactly the interstitial cells of the testicle. There is, too, a large quantity of fat which stains well with Sudan III.

A section through the hilum of the ovo-testis shows that the epoophoron or its homologue is present, although the structure is somewhat unusual; irregular tubules of large dimensions and containing secretions are seen cut across. These tubules are lined with several layers of polyhedral cells with prominent round nuclei. In places there are giant cells in the walls of the tubules beneath the epithelium, and they are especially well marked in the small intratubular projections which occur here and there. The nature of these giant cells is doubtful. They are probably due to the irritation of the secretion of the tubules. Bell thinks they are similar in nature to the giant cells which may be seen in the walls of the ovarian dermoids.

The masses and columns of cells in the central portion of the ovo-testis, which do not form tubules, resemble what may be seen in the early stage in the development of the normal testis; they have none of the appearance of malignant cells, for their nuclei are regular and quiescent, and the cells themselves are nearly all of one size and appear stable in their mode of growth.

With regard to the interstitial cells, it might be thought that they were ovarian and not testicular. As a rule, however, the interstitial cells of the ovary are much smaller, and never have I seen them so well marked, either in normal or pathological circumstances, as here shown. Further, if these interstitial cells were really ovarian one would expect to find them in the ovarian portion of the ovo-testis, where they are entirely absent, rather than in the testicular portion.

This is the general picture presented by an ovo-testis, but in the histological diagnosis of the condition there must always be difficulty. In the first place, there are masses of columnar cells which have no definite tubular arrangement. It was probably this irregularity of distribution which gave rise to the opinion already alluded to, that in this case the growth was malignant. In some parts, of course, there is a definitely tubular arrangement of the darkly staining columnar cells; and in other places tubules may be found lined with a single layer of epithelium, and containing secretion. There is, however, never any spermatogenesis to be seen.

It is particularly interesting to note in regard to the case recorded above, that the patient commenced life and passed puberty as a normal girl, menstruating regularly for eighteen months.; that menstruation had then ceased and masculine characteristics developed and that in spite of this she suffered from menopausal symptoms after operation. Nine months after operation, a complete change is to be noted in "her" appearance: the moustache has fallen out, all the hair on the legs has vanished, the voice is less deep, the skin less coarse, and her figure generally is much more feminine in regard to plumpness and outline. This, surely is dual sex characterization if ever there were such a thing.

With regard to the occurrence of the gonadal elements of the two sexes in one induvidual, one would naturally infer, from a developmental point of view, that the different elements would be combined in one organ.

In the earliest stages of development—that is to say, until about the thirtieth day—the histological appearances of the gonads give us no indication as to the future sex differentiation about to take place in these organs. In this undifferentiated stage the gonad is divided into two portions; the capsular epithelium, and the central epithelial nucleus composed of "indifferent" cells. Subsequently, however, if a testis is to be evolved, very soon after this date the cells of the epithelial nucleus immediately underlying the capsular epithelium become condensed and form the tunica albuginea, while the rest of these "indifferent" cells, among which the genital cells lie, become arranged in the form of cell masses or cords. These cords, around which and in which the spermatogonia—as the genital cells are now called—are collected, become the seminal tubules and eventually join the genital tubules outside the gonad· The interstitial cells of the testis are formed from connective tissue cells (mesoderm), which grow in from the direction of the hilum and fill the interstices between the seminal tubules. We shall see presently that the interstitial cells cannot originate from primary or secondary genital cells.

If, on the other hand, an ovary is in process of formation, the "indifferent" cells (epithelial nucleus) of the genital cell mass remain undifferentiated longer than in the case of testicular development, and they never become arranged in cell masses or cords. Instead, the epithelial nucleus becomes broken up by branching septa of ingrowing connective tissue, which divide the ovary into a meshwork of compartments. The tunica albuginea is formed by the meeting of these septa beneath the capsular epithelium. The indifferent cells enclosed in the connective tissue meshwork are believed to form secondary oogonia, but ultimately most of these degenerate and the spaces they previously occupied in the meshwork of connective tissue become filled by ingrowths from the septa. The interstitial cells of the ovary, like those of the membrana granulosa surrounding the surviving ova, are derived from the connective tissue stroma. In these circumstances, of course, the genital tubules atrophy, and their remains may be recognized, extending from the hilum of the ovary through the mesovarium into the mesosalpinx, as the epoophoron.

We can understand then, that if there be any hesitancy in the primary determination of sex the seminal tubules may commence to develop in the epithelial nucleus, even though they do not become functional in the ordinary sense of the word; and that the testicular interstitial cells may develop around them. One great difficulty, so it appears to us, and one which requires further elucidation, is why an ovo-testis may be found on one side in these circumstances and not on the other. Nevertheless, the plan of development described above almost negatives the possibility of the development of a separate ovary and testis on the same side: for it is extremely difficult to understand how one part of the genital cell mass could separate itself from the other and develop into a testis, while the remaining portion developed into an ovary.

It would seem, therefore, almost inevitable that, when there is dual characterization in the gonads, the only possible combination must be a testicular central portion surrounded by an ovarian. Bell lays down the following as essential conditions which must be established before any case can be considered one of glandular partial hermaphroditism:

(1) The hermaphroditic gonad must be an ovo-testis, composed of ovarian tissue with definite primordial ova, Graafian follicles or corpora albicantia, surrounding a central portion containing seminal tubules and testicular interstitial cells.

(2) The subject must show in the primary or secondary characteristics, other than the sex glands, evidences of hermaphroditism.

If these conditions be considered critical very few cases, probably only three (von Salen's Garre's, and Blacker's and Lawrence's), apart from Bell's, would pass the test.

